# Neuroticism and poor cardiovascular health may signal early cognitive dysfunction related to Alzheimer’s risk in older African Americans

**DOI:** 10.1002/alz.095731

**Published:** 2025-01-09

**Authors:** Ashley Powell, Bernadette A. Fausto, Mark A. Gluck

**Affiliations:** ^1^ Rutgers University–Newark, Newark, NJ USA

## Abstract

**Background:**

African Americans bear a disproportionate burden of both cardiovascular diseases and Alzheimer’s disease (AD). Assessment of the Five‐Factor personality dimensions–Neuroticism, Extroversion, Openness, Agreeableness, and Conscientiousness, particularly, higher levels of neuroticism–is linked to greater adverse cardiovascular and cognitive outcomes, including AD. Generalization–the ability to apply prior learning to new circumstances–is presented as a putative cognitive marker for the earliest stages of preclinical AD. However, the relationship between personality, cardiovascular health, and cognition is understudied in older African Americans. This study explored the relationship between personality, cardiovascular health, and cognitive function in a cohort of older African Americans.

**Method:**

83 cognitively healthy older African American participants from the Pathways to Healthy Aging in African Americans cohort study (M_age_ = 68.34 years, SD = 6.99; M_education_ = 13.93 years, SD = 2.36; M_MMSE_ = 27.65, SD = 1.95) provided a blood sample for blood sugar levels (HbA1c); participated in anthropometric/physiological measurements (e.g., body mass index and resting heart rate); completed an aerobic fitness assessment (six‐minute walk test for VO2max estimate); completed a cognitive battery including a generalization task (Concurrent Discrimination and Transfer Task) and neuropsychological tests; and responded to mood and demographic questionnaires. We binarized each personality dimension at the median creating a lower and higher group. Independent sample t‐tests and correlations were performed to assess the relationship between each of the personality dimensions, cardiovascular health, and cognitive variables.

**Result:**

Individuals in the higher neuroticism group had higher resting heart rate (d = ‐0.45), HbA1c levels (d = ‐0.58), and depressive symptomology (d = ‐0.80) while also demonstrating poorer aerobic fitness (d = 0.58) than their lower neuroticism counterparts. Subsequently, higher HbA1c was associated with poorer performance on the generalization task: Concurrent Discrimination and Transfer Task training phase (r = ‐.343, p = .002) and probe phase performance, (r = ‐.292, p = .008).

**Conclusion:**

We identified that cognitive function, specifically the ability to generalize prior learning is indirectly related to poor cardiovascular health in individuals that have a higher level of neuroticism. Our findings suggest that in older African American populations, the propensity to experience negative emotions (e.g., worry or any of the other neurotic traits) may negatively impact cardiovascular health, which may indirectly lead to poor cognitive outcomes such as dysfunction and AD.

